# Identification of ANT2 as a Druggable Target for Endocrine-Resistant ERα-Positive Breast Cancer

**DOI:** 10.3390/ijms27083704

**Published:** 2026-04-21

**Authors:** Erika Iguchi, Motoki Watanabe, Kaito Kobayashi, Shogen Boku, Wataru Nishio, Chikage Kato, Midori Morita, Koichi Sakaguchi, Michihiro Mutoh, Tomoshi Kameda, Yasuto Naoi

**Affiliations:** 1 Department of Endocrine and Breast Surgery, Kyoto Prefectural University of Medicine, 465 Kajii-cho Kawaramachi-Hirokoji, Kamigyo-ku, Kyoto 602-8566, Japan; erika628@koto.kpu-m.ac.jp (E.I.); c-kato@koto.kpu-m.ac.jp (C.K.); midori@koto.kpu-m.ac.jp (M.M.); ksak@koto.kpu-m.ac.jp (K.S.); naoi@koto.kpu-m.ac.jp (Y.N.); 2 Department of Molecular-Targeting Prevention, Kyoto Prefectural University of Medicine, 465 Kajii-cho Kawaramachi-Hirokoji, Kamigyo-ku, Kyoto 602-8566, Japan; mb211061@stu.kpu-m.ac.jp (W.N.); mimutoh@koto.kpu-m.ac.jp (M.M.); 3 Artificial Intelligence Research Center, National Institute of Advanced Industrial Science and Technology (AIST), 2-4-7 Aomi, Koto-ku, Tokyo 135-0064, Japan; k-kobayashi@aist.go.jp; 4 Department of Clinical Oncology, Kansai Medical University Hospital, 2-3-1, Shinmachi, Hirakata 573-1191, Japan; bokush@hirakata.kmu.ac.jp

**Keywords:** ERα-positive breast cancer, endocrine resistance, perillyl alcohol, adenine nucleotide translocase 2 (ANT2), molecular dynamics simulation, drug repurposing

## Abstract

Endocrine therapy is the mainstay for estrogen receptor (ER) α-positive breast cancer (BC), yet many patients display acquired resistance. We then screened natural compounds using human ERα-positive BC cells and identified perillyl alcohol (POH), a monoterpene from perilla, that reduces ERα protein levels. Chemoproteome analysis using POH-immobilized nanomagnetic beads revealed adenine nucleotide translocase 2 (ANT2), a mitochondrial inner membrane protein, as a direct target of POH. Molecular dynamics (MD) simulations predicted POH binding to the central pore of ANT2, which functions in ATP transport. ANT2 depletion reduced ERα levels, and public datasets indicate that high ANT2 expression correlates with poor prognosis in ERα-positive BC. POH also inhibited the growth of Tamoxifen- and Fulvestrant-resistant BC cells. RNA sequencing showed that fatty acid elongation-related genes were upregulated in Fulvestrant-resistant cells but downregulated by ANT2 depletion. Both ANT2 depletion and POH treatment led to the accumulation of intracellular lipid droplets in Fulvestrant-resistant cells, consistent with impaired fatty acid elongation. Finally, in silico screening using MD simulations identified venetoclax and nystatin as potential ANT2 pore binders. Both compounds reduced ERα levels in ERα-positive BC cells and increased lipid droplet formation in Fulvestrant-resistant cells. These findings highlight ANT2 as a druggable target against endocrine-resistant BC.

## 1. Introduction

Breast cancer (BC) remains the most commonly diagnosed malignancy among women worldwide, with more than 2.3 million new cases and 680,000 deaths reported in 2022 [1]. The global burden continues to rise, and in Asia specifically, the incidence is projected to reach 1.4 million new cases with 500,000 deaths by 2050 [2]. Approximately 70% of BC are estrogen receptor (ER)-positive and HER2-negative [3]. Endocrine therapy has long been established as the standard treatment for ER-positive BC. Selective estrogen receptor modulators (SERMs), such as Tamoxifen, exert anti-tumor effects by competing for ER binding, whereas selective estrogen receptor degraders (SERDs), such as Fulvestrant, downregulate ERα expression. Tamoxifen has been the mainstay of endocrine therapy for decades [4]. However, approximately 40% of patients treated with adjuvant Tamoxifen ultimately experience recurrence, and many patients with metastatic BC who initially respond to endocrine therapy eventually develop resistance [5]. Moreover, although Fulvestrant is widely used as a second-line option, resistance has also been reported in both metastatic and recurrent settings, often limiting its long-term efficacy [6]. These clinical observations indicate that endocrine resistance arises not only with SERMs but also with SERDs, underscoring the need to elucidate the diverse mechanisms underlying this phenomenon. To date, several pathways have been implicated in endocrine resistance, including mutations in *ESR1* that lead to constitutively active ERα [7], activation of growth factor receptor signaling pathways such as HER2 [8] and EGFR [9], and alterations in downstream PI3K/AKT/mTOR signaling [10,11]. Despite these insights, the molecular basis of endocrine resistance remains incompletely understood, underscoring the urgent need for novel therapeutic strategies.

Natural compounds represent a valuable reservoir for drug discovery. Among them, perillyl alcohol (POH), a monoterpene derived from plants such as *Perilla frutescens*, has attracted significant attention. POH exerts diverse antitumor effects across multiple cancer cell types, including induction of apoptosis [12,13], inhibition of Ras signaling [14,15], modulation of cell cycle regulators [13,16], and suppression of angiogenesis [17], prompting several early-phase clinical trials. In ER-positive BC, POH has also been shown to inhibit cell proliferation in preclinical studies [18,19]; however, its precise molecular targets and mechanisms of action remain unclear.

In this study, we employed a chemoproteomics approach and identified adenine nucleotide translocase 2 (ANT2) as a novel binding protein of POH. ANT2 is a mitochondrial inner membrane protein that mediates ATP/ADP exchange, and its aberrant expression has been implicated in the promotion of tumor growth by enhancing glycolysis [20] and inhibiting apoptosis [21]. Thus, although ANT2 has been linked to oncogenesis, its role in ER-positive BC and endocrine resistance remains unclear.

Here, using chemoproteomics, we demonstrate that POH exerts antitumor activity against ER-positive BC cells, including endocrine-resistant cells, by directly targeting ANT2, thereby uncovering a novel ANT2-dependent mechanism of endocrine resistance. Furthermore, through cheminformatics-based in silico screening, we identified clinically available compounds as candidate ANT2 inhibitors, highlighting a promising therapeutic strategy to overcome endocrine resistance in ER-positive BC.

## 2. Results

### 2.1. (S)-(-)-Perillyl Alcohol (POH) Suppresses Cell Growth of Estrogen Receptor (ER)-Positive Breast Cancer (BC) Cells with a Reduction in ER Expression

**Figure 1:**
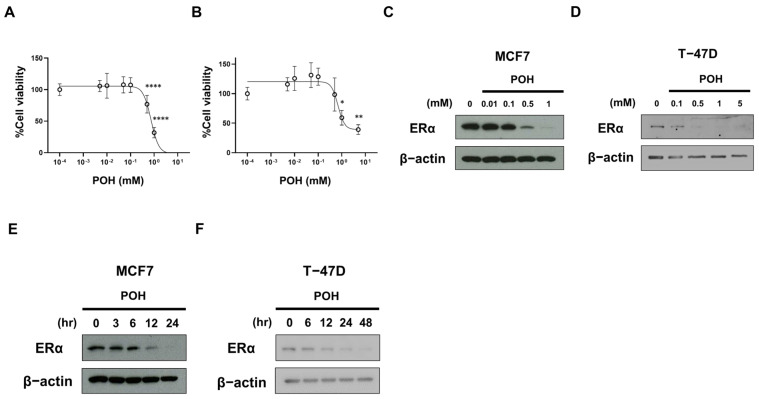
(S)-(-)-perillyl alcohol (POH) suppresses cell growth of estrogen receptor (ER)-positive breast cancer (BC) cells with a reduction in ER expression. (**A**,**B**) Analysis of cell growth of ER-positive BC cells after the treatment of POH. MCF7 (**A**) and T-47D (**B**) cells were treated with POH at the indicated concentrations for 120 h (**A**) or 96 h (**B**). Cell viability was measured with a Cell Counting Kit-8 assay. The data obtained with dimethyl sulfoxide (DMSO) were taken as 100%. Points, means (*n* = 4 in (**A**), *n* = 3 in (**B**)); bars, SD. * *p* < 0.05; ** *p* < 0.01; **** *p* < 0.0001, significantly different from the DMSO-treated control. (**C**,**D**) Analysis of the expression of ERα protein after the dose-dependent treatment of POH. ERα expression was analyzed by Western blotting in MCF7 (**C**) and T-47D (**D**) cells treated with POH at the indicated concentrations for 24 h (**C**) or 48 h (**D**). β-Actin was used as a loading control. (**E**,**F**) Analysis of the expression of ERα protein after the time-dependent treatment of POH. ERα expression was analyzed by Western blotting in MCF7 (**E**) and T-47D (**F**) cells treated with POH at 1 mM for the indicated times. β-Actin was used as a loading control.

To investigate the tumor-suppressive effect of POH on ER-positive BC cells, we evaluated the growth of the ER-positive human BC cell lines MCF7 and T-47D treated with POH. POH treatment significantly inhibited cell growth in a dose-dependent manner in both cell lines (Figure 1A,B). We next examined ERα protein expression after POH treatment and found that POH reduced ERα expression in a dose-dependent manner in both MCF7 (Figure 1C) and T-47D cells (Figure 1D). Furthermore, time-course analysis showed that ERα expression decreased over time following treatment with 1 mM POH (Figure 1E,F).

### 2.2. POH-Binding Protein Adenine Nucleotide Translocase 2 (ANT2) Is Involved in the Regulation of ERα Expression

**Figure 2:**
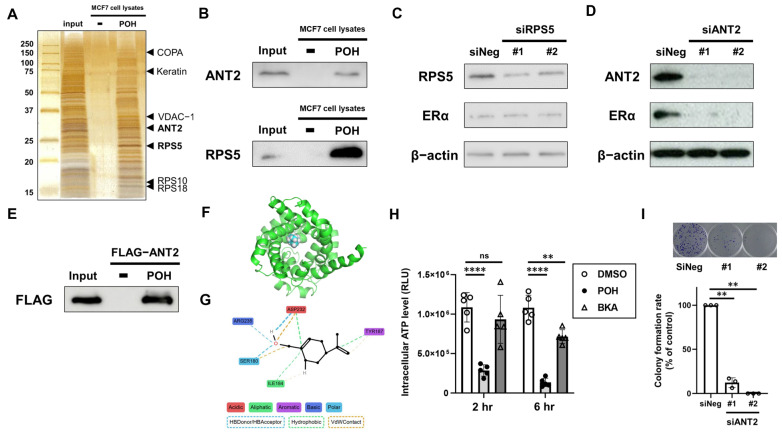
POH-binding protein ANT2 affects ER expression and growth of ER-positive BC cells. (**A**) Silver staining of POH-binding proteins. Purified POH-binding proteins from whole-cell extracts of MCF7 cells incubated with POH-immobilized FG beads were detected and identified using mass spectrometry. (**B**) Confirmation of the interaction of POH with RPS5 and ANT2. Bound RPS5 and ANT2 were detected by Western blotting with anti-RPS5 and anti-ANT2 antibodies, respectively. (**C**,**D**) Effects of RPS5 or ANT2 depletion on ERα expression. ERα expression was analyzed by Western blotting in MCF7 cells treated with negative control siRNA (siNeg) or siRNAs targeting different sequences (#1 and #2) of RPS5 (**C**) and ANT2 (**D**) gene for 48 h. β-Actin was used as a loading control. (**E**) Confirmation of the direct binding of POH with the recombinant ANT2 protein. Purified recombinant FLAG-ANT2 was incubated with POH-immobilized FG beads, and bound FLAG-ANT2 was detected by Western blotting with the anti-FLAG antibody. (**F**) The most stable docking pose of POH with ANT2 evaluated by the MD simulation and the scoring function of Smina. (**G**) Interaction network between ANT2 amino acid residues and POH. Interaction analysis was performed using ProLIF. For visual clarity, the original ProLIF-generated figure was modified. HB, hydrogen bond; VdW, van der Waals contact. (**H**) Effects of POH and bongkrekic acid (BKA) on intracellular ATP levels in ER-positive BC cells. MCF7 cells were treated with 1 mM POH, 100 µM BKA, or DMSO for 2 h and 6 h. Intracellular ATP levels were measured using the CellTiter-Glo^®^ 2.0 Cell Viability Assay. Points, means (*n* = 5); bars, SD. ns, not significant; ** *p* < 0.01; **** *p* < 0.0001. (**I**) Effects of ANT2 depletion on colony formation of ER-positive BC cells. MCF7 cells were treated with siNeg or siANT2 (#1 and #2) for 16 days. Colonies were fixed and stained with crystal violet. Representative images of stained colonies are shown (upper panel). Colony formation rates are shown in the graph (lower panel). Bars, means (*n* = 3). ** *p* < 0.01.

To investigate how POH suppresses ERα expression, we immobilized POH onto nanomagnetic beads (Figure S1) to identify its target proteins in ERα-positive BC cells. After incubating the beads with whole-cell lysates from MCF7 cells, POH-binding proteins were purified and detected by silver staining (Figure 2A). We then subjected each protein band after in-gel digestion to matrix-assisted laser desorption/ionization time-of-flight mass spectrometry (MALDI-TOF MS). We identified seven POH-binding proteins (Figure 2A), focusing on the mitochondrial inner membrane protein adenine nucleotide translocase 2 (ANT2) and the ribosomal protein S5 (RPS5), both of which have previously been reported to be oncogenic [22,23]. We then confirmed that POH interacts with both ANT2 and RPS5 in MCF7 cell lysates using specific antibodies of ANT2 and RPS5 (Figure 2B). Next, to determine which of these proteins regulates ERα expression, we performed siRNA knockdown experiments targeting ANT2 and RPS5. Whereas RPS5 depletion did not affect ERα expression (Figure 2C), ANT2 depletion led to a marked reduction in ERα expression (Figure 2D), prompting us to focus on ANT2 in subsequent analyses. We then confirmed that recombinant FLAG-tagged ANT2 protein bound to POH-immobilized beads (Figure 2E), indicating a direct interaction between POH and ANT2, while POH did not affect ANT2 expression levels (Figure S2). Molecular dynamics (MD) simulations were conducted to investigate the binding mode between ANT2 and POH. The simulations demonstrated that POH was tightly bound within the central pore of ANT2, which functions as a pathway for ATP translocation [24] (Figure 2F, Video S1). The root-mean-square deviation (RMSD) between ANT2 and POH remained consistently low throughout the simulation period, indicating a strong and stable interaction (Figure S3). To further characterize the interaction between POH and ANT2, an interaction network analysis was performed, revealing that POH interacts with five amino acid residues within the binding pocket of ANT2, including ARG235, ASP232, SER180, ILE184, and TYR187, through hydrogen bond donor and acceptor interactions, as well as hydrophobic interactions and van der Waals contacts. (Figure 2G). We then investigated whether POH affects cellular energy homeostasis via ANT2 inhibition by measuring intracellular ATP levels following POH treatment. As anticipated, POH reduced intracellular ATP levels, with a more rapid and pronounced decrease than that observed with the well-known ANT2 inhibitor, bongkrekic acid (BKA) (Figure 2H). Finally, ANT2 depletion suppressed the colony formation of MCF7 cells (Figure 2I). These results suggest that POH directly targets ANT2 and inhibits its function in ERα-positive BC cells.

### 2.3. ANT2 Expression Is Correlated with Clinical Aggressiveness in ERα-Positive BC

**Figure 3:**
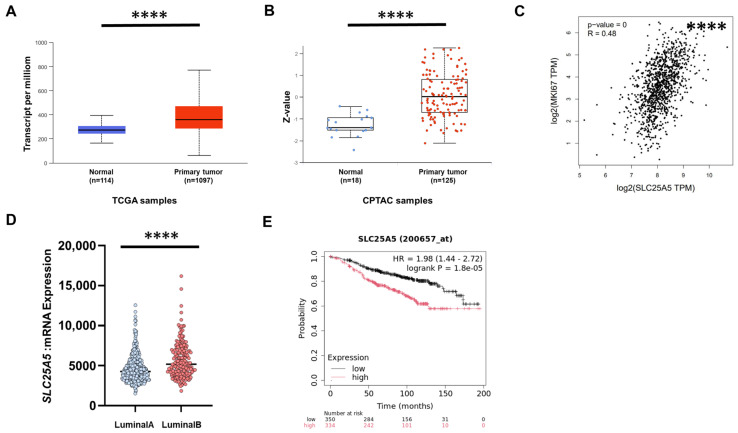
ANT2 expression is correlated with clinical aggressiveness in ERα-positive BC. (**A**) The comparison of *SLC25A5* mRNA expression between BC and normal tissue using UALCAN (http://ualcan.path.uab.edu (accessed on 14 April 2025)). **** *p* < 0.0001. (**B**) The comparison of ANT2 protein expression between BC and normal tissue using UALCAN. **** *p* < 0.0001. (**C**) Correlation analysis between the expressions of *SLC25A5* and *MKI67* in the TCGA BC data using GEPIA (http://gepia.cancer-pku.cn/ (accessed on 25 June 2025)). **** *p* < 0.0001. (**D**) The comparison of *SLC25A5* expression between Luminal A and B subtypes based on the TCGA dataset using cBioPortal (https://www.cbioportal.org/ (accessed on 9 July 2025)). **** *p* < 0.0001. (**E**) Prognosis analysis of ER-positive BC patients treated with endocrine therapy. Patients were stratified by *SLC25A5* expression, and recurrence-free survival (RFS) was analyzed using Kaplan–Meier Plotter (https://kmplot.com/ (accessed on 30 June 2025)).

To investigate whether ANT2 plays a biological role in a clinical setting of ERα-positive BC, we conducted a bioinformatics analysis using publicly available datasets. The study using UALCAN (http://ualcan.path.uab.edu (accessed on 14 April 2025)) [25] revealed that the expression of *SLC25A5* (coding ANT2) was significantly elevated in BC tissues compared with normal breast tissues at both mRNA (*p* value = 1.62 × 10^−12^; Figure 3A) and protein levels (*p* value = 9.31 × 10^−12^; Figure 3B). We next analyzed TCGA BC data using GEPIA (http://gepia.cancer-pku.cn/ (accessed on 25 June 2025)) [26] and found that *SLC25A5* expression showed a moderate positive correlation with MKI67 (coding Ki-67, a marker of cell proliferation) (R = 0.48, *p* value < 1.0 × 10^−7^; Figure 3C). ERα-positive BC are classified into two molecular subtypes, Luminal A and Luminal B. Among them, Luminal B tumors exhibit greater proliferative potential, higher Ki-67 expression, a higher risk of recurrence, and poorer clinical outcomes than Luminal A tumors [27]. Consistent with this, using cBioPortal (https://www.cbioportal.org/ (accessed on 9 July 2025)) [28,29], we found that *SLC25A5* expression was significantly higher in Luminal B subtypes than in Luminal A subtypes (*p* value < 0.0001; Figure 3D). Furthermore, we assessed the prognostic impact of *SLC25A5* expression using the Kaplan–Meier Plotter (https://kmplot.com (accessed on 30 June 2025)) [30] and found that high *SLC25A5* expression was significantly associated with worse relapse-free survival (RFS) of ER-positive and HER2-negative BC patients (HR = 1.98; 95% CI = 1.44–2.72; *p* value = 1.8 × 10^−5^) (Figure 3E). By contrast, high expression of another POH-binding protein, RPS5, was not associated with prognosis (RFS: HR = 0.78; 95% CI = 0.57–1.06; *p* value = 0.11) (Figure S4). Taken together, these findings suggest that ANT2 is associated with the clinical aggressiveness of ER-positive BC.

### 2.4. POH Suppresses Cell Growth in Endocrine-Resistant Cells

**Figure 4:**
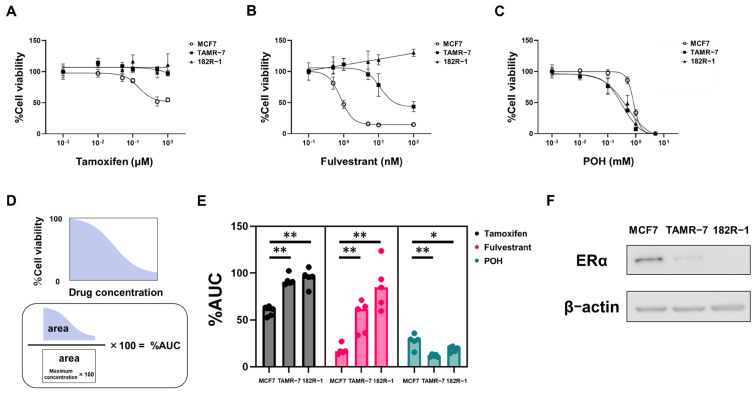
POH suppresses cell growth in endocrine-resistant cells. (**A**–**C**) Analysis of cell growth of MCF7, TAMR-7, and 182R-1 cells after the treatment of Tamoxifen, Fulvestrant, and POH. MCF7, TAMR-7, and 182R-1 cells were treated with Tamoxifen (**A**), Fulvestrant (**B**), and POH (**C**) for 120 h. Cell viability was measured with a Cell Counting Kit-8 assay. The data obtained with dimethyl sulfoxide (DMSO) were taken as 100%. Points, means (*n* = 4); bars, SD. (**D**) %AUC was defined as the percentage obtained by dividing the area under the growth inhibition curve (AUC) by the total area corresponding to 100% viability at the highest concentration (AUC × 100). %AUC was calculated using GraphPad Prism (v9.5.1), G. (**E**) The %AUC of Tamoxifen, Fulvestrant, and POH against MCF7, TAMR-7, and 182R-1 cells. Bars, means. Data from five replicate experiments are shown. * *p* < 0.05, ** *p* < 0.01. (**F**) The comparison between ERα protein expression of MCF7, TAMR-7, and 182R-1 cells. ERα expression was analyzed by Western blotting in each cell. β-Actin was used as a loading control.

Next, we examined whether POH exhibits antitumor effects against endocrine-resistant cells using MCF7/TAMR-7 (hereafter referred to as TAMR-7) and MCF7/182R-1 (hereafter referred to as 182R-1), which are MCF7-based resistant cell lines to the conventional anti-estrogen agents Tamoxifen and Fulvestrant, respectively. Expectedly, Tamoxifen inhibited the growth of parental MCF7 cells but had minimal effect on both TAMR-7 and 182R-1 cells (Figure 4A), while Fulvestrant was effective in MCF7 and TAMR-7 cells but not in 182R-1 cells (Figure 4B). In contrast, POH suppressed cell growth not only in MCF7 cells but also more potently in TAMR-7 and 182R-1 cells (Figure 4C). We further calculated the percentage of area under the curve (%AUC), as defined in Figure 4D, and clearly demonstrated that POH was even more effective in these resistant cells (Figure 4E). Notably, ERα expression was apparently reduced in TAMR-7 and 182R-1 cells compared with MCF7 cells (Figure 4F), suggesting that an ERα-independent mechanism is involved in endocrine resistance.

### 2.5. The Fatty Acid Elongation Pathway Is Involved in Fulvestrant-Resistant Cells

**Figure 5:**
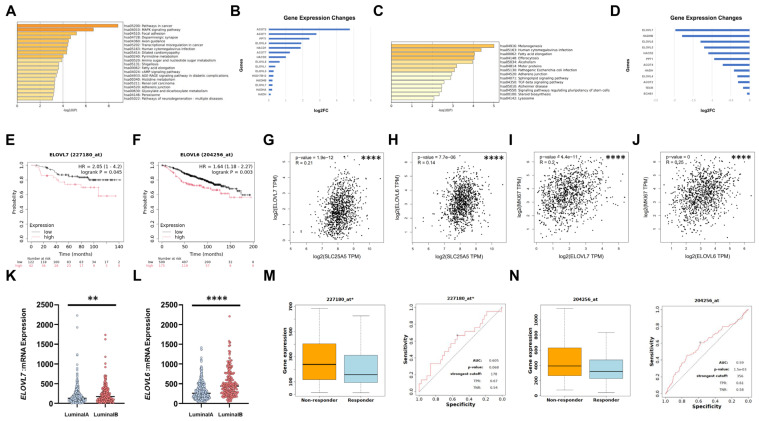
The fatty acid elongation pathway is involved in Fulvestrant-resistant cells. (**A**) The top 20 pathways of enrichment analysis of upregulated genes in 182R-1 cells compared with MCF7 cells based on RNA-seq data. (**B**) The comparison of fatty acid elongation-related DEGs between MCF7 and 182R-1 cells based on RNA-seq data. (**C**) The top 14 pathways of enrichment analysis of downregulated genes upon ANT2 depletion by siANT2 (#2) in 182R-1 cells based on RNA-seq data. (**D**) The comparison of fatty acid elongation-related DEGs with or without ANT2 depletion by siANT2 (#2) in 182R-1 cells based on RNA-seq data. (**E**,**F**) Prognosis analysis of ER-positive BC patients treated with endocrine therapy. Patients were stratified by *ELOVL7* (**E**) and *ELOVL6* (**F**) expression, and recurrence-free survival (RFS) was analyzed using the Kaplan–Meier Plotter (https://kmplot.com/ (accessed on 30 June 2025)). (**G**,**H**) Correlation analysis between the expressions of *SLC25A5* and *ELOVL7* (**G**) or *ELOVL6* (**H**) in the TCGA BC data using GEPIA (http://gepia.cancer-pku.cn/ (accessed on 25 June 2025)). **** *p* < 0.0001. (**I**,**J**) Correlation analyses between the expressions of *MKI67* and *ELOVL7* (**I**) or *ELOVL6* (**J**) in the TCGA BC data using GEPIA. **** *p* < 0.0001. (**K**,**L**) The comparison of *ELOVL7* (**K**) and *ELOVL6* (**L**) expression between Luminal A and B subtypes based on the TCGA dataset using cBioPortal (https://www.cbioportal.org/ (accessed on 9 July 2025)). ** *p* < 0.01, **** *p* < 0.0001. (**M**,**N**) ROC curve analysis of *ELOVL7* (**M**) and *ELOVL6* (**N**) expression in responder and non-responder groups to endocrine therapy in ER-positive BC using the ROC Plotter (https://rocplot.com/ (accessed on 28 May 2025)).

To elucidate the mechanism of endocrine resistance, we performed RNA-seq analysis to identify differentially expressed genes (DEGs) in two comparisons: (i) Fulvestrant-resistant 182R-1 cells versus parental MCF7 cells (Figure S5), and (ii) ANT2-depleted 182R-1 cells versus negative control cells (Figure S6). Interestingly, both gene sets showed a common enrichment in genes involved in the fatty acid elongation pathway (Figure 5A–D). Among these fatty acid elongation-related genes, we focused on the top three downregulated genes upon ANT2 knockdown: *ELOVL7*, *HADHB*, and *ELOVL6*. *ELOVL7* (HR = 2.05; 95% CI = 1.00–4.20; *p* value = 0.045) (Figure 5E) and *ELOVL6* (HR = 1.64; 95% CI = 1.18–2.27; *p* value = 0.003) (Figure 5F) were significantly associated with poor prognosis in patients with ER-positive and HER2-negative BC treated with endocrine therapy, whereas *HADHB* (HR = 1.17; 95% CI = 0.84–1.64; *p* value = 0.35) showed no such association (Figure S7). We then further analysed the expressions of *ELOVL7* and *ELOVL6* and found weak positive correlations between these genes and *SLC25A5* levels (*ELOVL7*: R = 0.21, *p* value = 1.9 × 10^−12^; *ELOVL6*: R = 0.14, *p* value = 7.7 × 10^−6^) (Figure 5G,H), as well as with MKI67 levels (*ELOVL7*: R = 0.20, *p* value = 1.4 × 10^−11^; *ELOVL6*: R = 0.25, *p* value < 1.0 × 10^−7^) (Figure 5I,J) in the TCGA breast cancer dataset. Furthermore, both *ELOVL7* and *ELOVL6* genes were significantly upregulated in the Luminal B subtype compared with the Luminal A subtype (*p* value < 0.01, *p* value < 0.0001) (Figure 5K,L) in non-responders to endocrine therapy in ER-positive and HER2-negative BC (AUC = 0.605; *p* value = 0.068, AUC = 0.59; *p* value = 1.5 × 10^−3^) (Figure 5M,N). These results suggest that the expression of a subset of fatty acid elongation-related genes is associated with the clinical aggressiveness of ER-positive BC, including resistance to endocrine therapy.

### 2.6. Targeting ANT2 Leads to Lipid Droplet Accumulation in Fulvestrant-Resistant Cells

**Figure 6:**
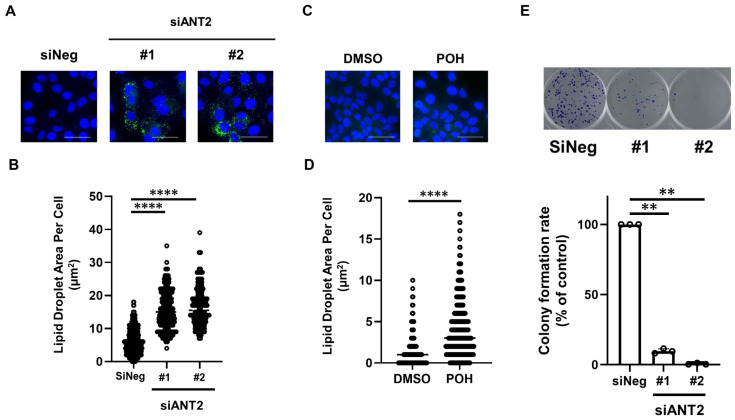
Targeting ANT2 leads to lipid droplet accumulation in Fulvestrant-resistant cells. (**A**–**D**) Effects of ANT2 depletion and POH treatment on lipid droplet accumulation in Fulvestrant-resistant cells. 182R-1 cells were treated with negative control siRNA (siNeg) or siANT2 (#1 and #2) for 72 h (**A**,**B**) and 500 µM POH for 72 h (**C**,**D**). Cells were fixed and stained with ShoyakuGreen (green, lipid droplets) and Hoechst 33342 (blue, cell nuclei). Representative images of stained cells are shown (**A**,**C**). Scale bars, 5 µm. Lipid droplet area per cell is shown in the graph (**B**,**D**). **** *p* < 0.0001. (**E**) Effects of ANT2 depletion on colony formation of Fulvestrant-resistant cells. 182R-1 cells were treated with siNeg or siANT2 (#1 and #2) for 16 days. Colonies were fixed and stained with crystal violet. Representative images of stained colonies are shown (upper panel). Colony formation rates are shown in the graph (lower panel). Columns, means (*n* = 3); bars, SD. ** *p* < 0.01.

As the fatty acid elongation pathway (and possibly fatty acid utilization) has been implicated in endocrine resistance, we examined lipid droplet accumulation in ANT2-depleted Fulvestrant-resistant cells. ANT2 depletion significantly increased lipid droplet formation in 182R-1 cells (*p* value < 0.0001) (Figure 6A,B). Similarly, treatment with POH also enhanced lipid droplet accumulation in 182R-1 cells (*p* value < 0.0001) (Figure 6C,D). Furthermore, ANT2 depletion markedly suppressed the colony formation of 182R-1 cells (Figure 6E). These results suggest that targeting ANT2 suppresses fatty acid elongation, thereby promoting lipid droplet accumulation and inhibiting cell growth in Fulvestrant-resistant cells.

### 2.7. In Silico Drug Screening Identified Candidate Repurposed Ligands for ANT2

**Figure 7:**
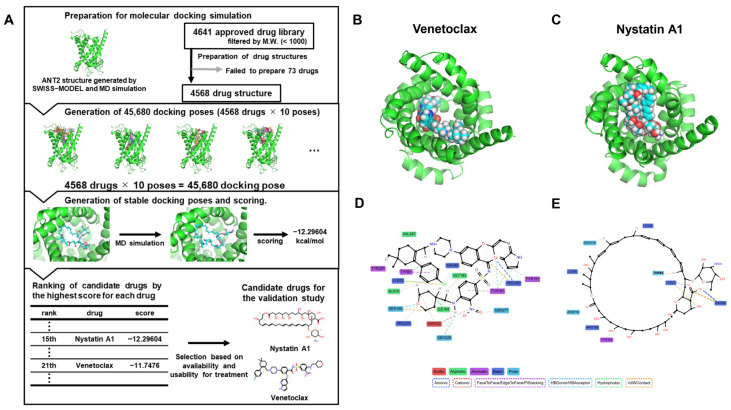
In silico drug screening identified candidate repurposed ligands for ANT2. (**A**) Workflow of in silico screening for ANT2 ligands (see Materials and Methods section for details). (**B**,**C**) The most stable docking pose of Venetoclax (**B**) and Nystatin A1 (**C**) with ANT2 evaluated by the MD simulation and the scoring function of Smina. (**D**,**E**) Interaction networks between amino acid residues and Venetoclax (**D**) or Nystatin A1 (**E**). The interaction analyses were performed using ProLIF. For visual clarity, the figures output by ProLIF were modified. HB, hydrogen bond; VdW, van der Waals contact.

To discover more effective and clinically feasible ANT2 ligands, we performed in silico screening among FDA-approved drugs. The predicted ANT2 structure was used for molecular docking calculations, which were performed by Smina software (15 October 2019 version) [31] to generate complex structures. The docking grid was defined around the ATP/ADP-binding pocket of ANT2 [24], and 10 docking poses were generated for each drug. Then, MD simulation was performed to relax the docking pose and select the most stable pose based on the binding affinity predicted by the Smina scoring function [31]. The candidate drugs were ranked according to scores predicted by the Smina scoring function [31], and the list was then narrowed based on availability and clinical usability. Among the hit compounds, we found the Bcl-2 inhibitor Venetoclax ranked 21st, and Nystatin A1 (the main component of the antifungal agent Nystatin) ranked 15th (Figure 7A). We performed MD simulations again, repeating the 20 ns simulation at 300 K 10 times, to confirm the stability of the docking poses of Venetoclax (Figure 7B, Video S2) and Nystatin A1 (Figure 7C, Video S3). The RMSD between ANT2 and Venetoclax (Figure S8) and Nystatin A1 (Figure S9) showed that these compounds stably bind to ANT2 over time. Interaction network analysis revealed that Venetoclax interacts with multiple ANT2 residues through a variety of noncovalent interactions, including hydrogen bond donor and acceptor interactions, hydrophobic interactions, and additional noncovalent interactions, such as π-related and electrostatic contacts (Figure 7D). Nystatin also exhibited multiple interaction types, with fewer interacting residues than those of Venetoclax (Figure 7E).

### 2.8. Venetoclax and Nystatin Exhibit Tumor-Suppressive Effects Against ER-Positive BC, Including Endocrine-Resistant Cells

**Figure 8:**
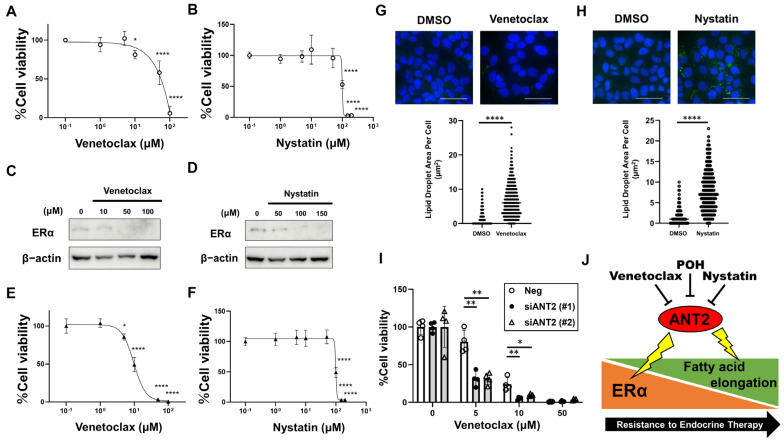
Venetoclax and Nystatin exhibit tumor-suppressive effects against ER-positive BC, including endocrine-resistant cells. (**A**,**B**) Analysis of cell growth of ER-positive BC cells after the treatment of ANT2 ligands. MCF7 cells were treated with Venetoclax (**A**) and Nystatin (**B**) at the indicated concentrations for 120 h. Cell viability was measured with a Cell Counting Kit-8 assay. The data obtained with dimethyl sulfoxide (DMSO) were taken as 100%. Points, means (*n* = 4); bars, SD. * *p* < 0.05; **** *p* < 0.0001, significantly different from the DMSO-treated control. (**C**,**D**) Analysis of the expression of ERα protein after the dose-dependent treatment of ANT2 ligands. ERα expression was analyzed by Western blotting in MCF7 cells treated with Venetoclax (**C**) and Nystatin (**D**) at the indicated concentrations for 24 h. β-Actin was used as a loading control. (**E**,**F**) Analysis of cell growth of Fulvestrant-resistant cells after the treatment of ANT2 ligands. 181R-1 cells were treated with Venetoclax (**E**) and Nystatin (**F**) at the indicated concentrations for 120 h. Cell viability was measured with a Cell Counting Kit-8 assay. The data obtained with dimethyl sulfoxide (DMSO) were taken as 100%. Points, means (*n* = 4); bars, SD. * *p* < 0.05; **** *p* < 0.0001, significantly different from the DMSO-treated control. (**G**,**H**) Effects of ANT2 ligand treatment on lipid droplet accumulation in Fulvestrant-resistant cells. 182R-1 cells were treated with 10 µM Venetoclax (**G**) and 100 µM Nystatin (**H**) for 72 h. Cells were fixed and stained with ShoyakuGreen (green, lipid droplets) and Hoechst 33342 (blue, cell nuclei). Representative images of stained cells are shown (upper panel). Scale bars, 5 µm. Lipid droplet area per cell is shown in the graph (lower panel). **** *p* < 0.0001. (**I**) Effects of ANT2 depletion on Venetoclax sensitivity in Fulvestrant-resistant cells. 182R-1 cells were transfected with siNeg or siANT2 (#1 and #2). Venetoclax was added on day 3 at the indicated concentrations, and cell viability was measured on day 8. Cell viability was measured using a Cell Counting Kit-8 assay. For each group (siNeg, siANT2 (#1), and siANT2 (#2)), the value at 0 µM Venetoclax was set as 100%. Points, means (*n* = 4); bars, SD. * *p* < 0.05; ** *p* < 0.01, significantly different from the DMSO-treated control. (**J**) Schematic overview of endocrine resistance and its overcoming by ANT2 targeting in ER-positive BC.

To validate the proof of concept for targeting ANT2 via its ligands, we evaluated the tumor-suppressive effects of Venetoclax and Nystatin in ER-positive BC, including endocrine-resistant cells. Both Venetoclax and Nystatin significantly inhibited cell growth in MCF cells (Figure 8A,B), with dose-dependent reductions in ERα protein levels (Figure 8C,D). We further confirmed the effects of Venetoclax and Nystatin on 182R-1 cells. Venetoclax and Nystatin also significantly inhibited cell growth (Figure 8E,F). Additionally, these compounds increased lipid droplet accumulation in 182R-1 cells compared to DMSO controls (Figure 8G,H). Notably, ANT2 knockdown significantly enhanced the sensitivity of 182-R1 cells to Venetoclax treatment at 5–10 μM (Figure 8I), supporting a functional link between ANT2 status and Venetoclax responsiveness, and is compatible with the possibility that Venetoclax may, at least in part, function as an ANT2 inhibitor. Taken together, newly identified ANT2 ligands, Venetoclax and Nystatin, exhibit tumor-suppressive effects against ER-positive BC, including endocrine-resistant cells, consistent with results observed with POH treatment or ANT2 depletion.

## 3. Discussion

In this study, we employed an integrated chemoproteomics and cheminformatics approach and obtained the following findings: (i) the natural compound POH exerted antitumor effects against ERα-positive BC cells, including endocrine-resistant cells, by targeting ANT2; (ii) we uncovered a novel mechanism of endocrine resistance involving ANT2 and fatty acid elongation; and (iii) Venetoclax and Nystatin were identified as potential repurposable ANT2 ligands to overcome endocrine resistance (Figure 8J).

We first demonstrated that POH directly binds to ANT2 and suppresses ERα expression. ERα protein stability is maintained by ATP-dependent molecular chaperones such as HSP70 [32] and HSP90 [33], while ANT2 regulates cellular ATP homeostasis [34]. In line with this, we observed that POH treatment reduces intracellular ATP levels (Figure 2H), supporting this mechanistic link that inhibition or downregulation of ANT2 may reduce intracellular ATP availability, impair chaperone function, and ultimately destabilize ERα protein. The direct causal relationship between ANT2 function and ERα regulation remains to be fully elucidated and is currently under investigation.

Endocrine resistance has traditionally been attributed to genetic alterations, such as *ESR1* mutations [7], or the activation of kinase signaling pathways, including HER2 [8], EGFR [9], and PI3K/AKT/mTOR [10,11]. However, accumulating evidence has highlighted the role of metabolic rewiring, particularly lipid metabolism, in this resistance [35]. In this study, we found that endocrine resistance is linked to both ANT2 and the fatty acid elongation pathway. Because fatty acid elongation requires substantial ATP input, inhibition of ANT2 may impair ATP supply, thereby limiting elongase activity and restricting fatty acid utilization for cancer growth, ultimately leading to lipid droplet accumulation. To verify this hypothesis, lipidomic profiling and functional validation will be necessary in the future study. Importantly, we identified *ELOVL6* and *ELOVL7* as correlating with poor prognosis and clinical aggressiveness in ER-positive BC, including endocrine-resistant cases. Previous studies have shown that *ELOVL1*, *5*, and *6* are significantly elevated in ER-positive/HER2-negative and triple-negative BC compared with normal tissue [36]. High *ELOVL6* expression has been associated with poor prognosis in both breast [37] and liver [38] cancers, while *ELOVL7* has been implicated in prostate cancer progression through saturated long-chain fatty acid metabolism [39]. Moreover, elevated levels of saturated very long-chain fatty acids have been reported in BC tissue compared with normal tissue [40]. Collectively, these findings suggest that fatty acid elongation-related genes such as *ELOVL6* and *ELOVL7* may function not only as mechanistic mediators of endocrine resistance but also as predictive biomarkers for patient stratification.

POH has attracted attention as a natural compound with broad antitumor activity across multiple cancer types [41,42,43,44,45] in clinical studies; however, several limitations hinder its clinical application. The concentrations required to achieve activity in our experiments were in the millimolar range, and POH undergoes rapid metabolic conversion into aldehydes under physiological conditions [41], raising concerns regarding efficacy and safety. These limitations prompted us to search for clinically applicable ligands of ANT2. Using in silico drug screening based on MD simulations, we identified two repurposable candidates -Venetoclax and Nystatin. Proof-of-concept studies demonstrated that both agents reproduced the effects of POH or ANT2 depletion, including ERα downregulation in MCF7 cells and lipid droplet accumulation in 182-R1 cells.

The present study has several limitations and challenges that remain to be addressed. First, ANT2-ligand interactions were evaluated using chemical pull-down assays and MD simulations; however, more rigorous quantitative characterization using biophysical approaches, such as surface plasmon resonance, will be important in future studies. Second, Nystatin treatment caused an abrupt and pronounced reduction in cell viability (Figure 8B,F), raising concern that its effects are largely attributable to global membrane disruption [46] rather than to specific ANT2 inhibition. Nystatin primarily binds to ergosterol in fungal membranes; however, at higher concentrations, it can also interact with cholesterol-containing membranes, potentially leading to nonspecific membrane disruption. Therefore, the growth-inhibitory effect of Nystatin observed in this study should be interpreted with caution, as it may reflect nonspecific cytotoxicity rather than a specific ANT2-mediated effect. Furthermore, Nystatin is poorly absorbed from the gastrointestinal tract and unlikely to achieve therapeutic concentrations in breast tumors. Overcoming this limitation will require strategies such as drug-delivery systems or chemical modification to improve tissue penetration. Third, regarding Venetoclax, target specificity should be carefully considered, as it is an approved BCL-2 inhibitor, and its antitumor effects cannot be attributed solely to ANT2 modulation. We observed that ANT2 knockdown significantly enhanced the sensitivity of fulvestrant-resistant BC cells to Venetoclax (Figure 8I), supporting a functional link between ANT2 and Venetoclax responsiveness. Furthermore, previous studies have demonstrated that Venetoclax can suppress the growth of cancer cell lines with relatively low BCL-2 expression, such as the triple-negative BC cell line MDA-MB-231 [47] and the cervical cancer cell line HeLa [48], suggesting that its antitumor activity may not be fully explained by canonical BCL-2 inhibition alone. Taken together, these findings are consistent with the possibility that Venetoclax may, at least in part, act through ANT2 inhibition; however, this interpretation remains to be fully established and requires further mechanistic investigation. From a clinical standpoint, although previous phase I/II trials combining Venetoclax with Tamoxifen [49] or Fulvestrant [50] were negative, our findings suggest that biomarker-guided stratification based on ANT2 expression or fatty acid elongation signatures may help identify patients who could benefit from such combinations. Thus, Venetoclax, when combined with appropriate patient stratification, offers a promising approach to overcoming endocrine resistance.

In conclusion, our study highlights the potential of chemoproteomics to identify unique protein targets for cancer that may be undetectable by genomic-based approaches alone. Furthermore, integrating cheminformatics and computational structural biology enables rapid discovery of inhibitory ligands for target proteins, thereby bridging mechanistic insights with therapeutic exploration. By applying this integrated strategy, we identified ANT2 as a novel target of endocrine resistance in BC, elucidated its mechanistic link to lipid metabolic reprogramming, and provided proof-of-concept evidence that pharmacological inhibition of ANT2 may be a promising therapeutic strategy. Together, these findings suggest that our integrated chemoproteomics and cheminformatics approach may provide a broadly applicable platform that not only advances understanding of molecular mechanisms, including tumor growth, progression, and therapeutic resistance, through the identification of target proteins, but also facilitates the discovery of candidate drugs that target these mechanisms.

## 4. Materials and Methods

### 4.1. Cell Lines and Culture

Human breast cancer MCF7 cells were obtained from the NCI-60 cell lines of the NCI Developmental Therapeutics Program. T-47D cells were obtained from the American Type Culture Collection. The endocrine-resistant MCF7/TAMR-7 and MCF7/182R-1 cells were obtained from the European Collection of Authenticated Cell Cultures. Cell lines were confirmed to be free of mycoplasma contamination using the MycoAlert Mycoplasma Detection Kit (Lonza, Rockland, ME, USA). MCF7 cells were cultured in Dulbecco’s Modified Eagle’s Medium (DMEM) supplemented with 10% fetal bovine serum (FBS), 4 mM L-glutamine, 50 U/mL penicillin, and 100 μg/mL streptomycin. T-47D cells were cultured in RPMI-1640 supplemented with 10% FBS, 2 mM L-glutamine, 50 U/mL penicillin, and 100 μg/mL streptomycin. MCF7/TAMR-7 and MCF7/182R-1 cells were maintained in DMEM described above in the presence of 1 µM Tamoxifen and 100 nM ICI 182,780, respectively. All cell lines were incubated at 37 °C in a humidified 5% CO_2_ atmosphere.

### 4.2. Reagents

(S)-(-)-perillyl alcohol (POH) was obtained from Wako (Osaka, Japan). Tamoxifen was obtained from Sigma-Aldrich (Saint Louis, MO, USA). ICI 182,780 (Fulvestrant) was obtained from Tocris Cookson (Bristol, UK). Bongkrekic acid was obtained from LKT Laboratories (St. Paul, MN, USA). Venetoclax (ABT-199) and Nystatin were obtained from Selleck Biotech (Tokyo, Japan). These reagents were dissolved in dimethyl sulfoxide (DMSO) to prepare stock solutions, which were then diluted to the desired working concentrations in the culture medium.

### 4.3. Cell Viability Assay

The number of viable cells was measured using the Cell Counting Kit-8 assay (Dojindo, Kumamoto, Japan). Cells were seeded in 96-well plates at a density of 2000 cells per well for MCF7 cells, MCF7/TAMR-7 cells, and MCF7/182R-1 cells; 8000 cells per well for T-47D cells. After a 24 h incubation, the cells were treated with each reagent. Cells were then incubated with each agent for the indicated hours in the figure legends, and the kit reagent WST-8 was added to the medium and incubated for 4 h. Absorbance at 450 nm was measured using a multi-plate reader (Multiskan FC, Thermo Fisher Scientific, Waltham, MA, USA).

### 4.4. Protein Isolation and Western Blotting

Cells were lysed with a buffer containing 50 mM Tris-HCl [pH 8.0], 1% SDS, 1 mM dithiothreitol (DTT), and 0.43 mM 4-(2-aminoethyl) benzenesulfonyl fluoride hydrochloride (ABSF). The lysates were sonicated and centrifuged at 20,400 *g* for 20 min at 4 °C, and the supernatant was collected. Equal amounts of the protein extract were subjected to sodium dodecyl sulfate–polyacrylamide gel electrophoresis (SDS-PAGE) and transferred to a polyvinylidene difluoride membrane (EMD Millipore, Billerica, MA, USA). The following were used as the primary antibodies: mouse anti-human ERα monoclonal antibody (sc-8005; Santa Cruz Biotechnology, Inc., Dallas, TX, USA), rabbit anti-RPS5 (ab58345; Abcam, Cambridge, UK), rabbit anti-human ANT2 monoclonal antibody (#14671; Cell Signaling Technology, Beverly, MA, USA), mouse anti–β-actin monoclonal antibody (A5441; Sigma-Aldrich). Signals were detected with Chemi-Lumi One L (Nacalai Tesque, Kyoto, Japan) or Immobilon Western Chemiluminescent HRP Substrate (EMD Millipore).

### 4.5. Preparation of POH-Immobilized Beads

Immobilization of POH onto FG beads (TAS8848N1140, Tamagawa Seiki, Nagano, Japan) with carboxyl linkers was performed as previously described [51]. Briefly, the beads were incubated with N-hydroxysuccinimide (Peptide Institute, Osaka, Japan) and 1-ethyl-3-(3-dimethylaminopropyl)-carbodiimide hydrochloride (Nacalai Tesque) at 25 °C for 2 h to activate the carboxyl groups on the beads. Subsequently, POH was immobilized onto the beads in the presence of DMAP (Nacalai Tesque) by incubating overnight at 25 °C. Unreacted residues on the beads were masked with 2-aminoethanol (Nacalai Tesque) at 25 °C for 4 h. The final beads were stored at 4 °C.

### 4.6. Purification and Identification of POH-Binding Proteins

MCF7 cells were lysed in NP-40 lysis buffer (50 mM Tris-HCl [pH 8.0], 150 mM NaCl, 1% NP-40, 1 mM DTT, and 0.43 mM ABSF) by rotating at 4 °C for 30 min, then centrifuged at 20,400× *g* for 10 min at 4 °C. The supernatants were used as whole-cell extracts of MCF7 cells. The extracts were incubated with empty or POH-immobilized beads for 4 h at 4 °C. The beads were washed three times with a binding buffer (50 mM Tris-HCl [pH 8.0], 150 mM NaCl, and 0.1% NP-40). The proteins binding to the beads were eluted with Laemmli dye and subjected to SDS-PAGE. Then, POH-binding proteins were detected by silver staining. Each strip containing a POH-binding protein was cut out and digested with Sequencing Grade Modified Trypsin (Promega, Madison, WI, USA). The peptide fragments from each strip were analyzed using an Autoflex II mass spectrometer (Bruker Daltonics, Billerica, MA, USA), and each POH-binding protein was identified by peptide mass fingerprinting. Similarly, the recombinant FLAG-tagged ANT2 protein (TP308949; OriGene, Rockville, MD, USA) was incubated with empty or POH-immobilized beads for 4 h at 4 °C. The beads were washed three times with a binding buffer described above. FLAG-ANT2 bound to the beads was eluted with Laemmli dye, subjected to SDS-PAGE, and detected by Western blotting with a mouse anti-DDK (FLAG) monoclonal antibody (TA50011-100; OriGene).

### 4.7. RNAi

Oligonucleotides of siRNA targeting ANT2 and RPS5 were obtained from Invitrogen (Carlsbad, CA, USA): siANT2 #1 (HSS100497; Stealth siRNAs), siANT2 #2 (HSS179022; Stealth siRNAs), siRPS5 #1 (HSS109357; Stealth siRNAs), siRPS5 #2 (HSS184430; Stealth siRNAs), and a negative control siRNA (12,935,112; Stealth RNAi siRNA Negative Control Med GC Duplex #2). The sequences of these siRNAs are provided in Table S1. Cells were transfected with 10 nM siRNA using Lipofectamine RNAiMAX Reagent (Invitrogen) according to the manufacturer’s instructions.

### 4.8. Computational Evaluation of the Complex of ANT2 and the Drugs

The relaxed docking poses of POH, Venetoclax, and Nystatin A1 with ANT2 were used as the initial structures for the MD simulations. The 20 ns MD simulation at 300 K in water was repeated 10 times. The RMSD values were calculated by GROMACS tools (2022.4 version) in the following steps: (1) fitting the structures of each frame using Cα atoms, (2) calculating RMSD values using the heavy atoms of the drugs. The interaction networks between amino acid residues of the protein and the drugs across ten independent 20 ns MD simulations (total sampling of 200 ns) were analyzed using ProLIF ver. 2.1.0 [52].

### 4.9. ATP Assay

MCF7 cells were seeded at a density of 15,000 cells per well in 96-well plates. After a 24 h incubation, the cells were treated with 1 mM POH, 100 µM bongkrekic acid, or DMSO as a control for 2 h or 6 h. Intracellular ATP levels were determined using the CellTiter-Glo^®^ 2.0 Cell Viability Assay (Promega, Madison, WI, USA) according to the manufacturer’s instructions. Briefly, the plates and CellTiter-Glo^®^ 2.0 reagent were equilibrated to room temperature for 15 min before the assay. An equal volume of CellTiter-Glo^®^ 2.0 reagent, corresponding to the volume of culture medium present in each well, was added to each well, and the plates were incubated for 10 min at room temperature to stabilize the luminescent signal. Luminescence was measured using a luminometer (Navigator System GM2000, Promega).

### 4.10. Colony Formation Assay

Cells were seeded at a density of 500 cells per well in 6-well plates. After siRNA transfection, they were cultured for 16 days. The cells were then immobilized with 10% formalin and stained with 0.1% crystal violet. The areas of the stained colonies were quantified using ImageJ (v1.54g, NIH, Bethesda, MD, USA; https://imagej.net/software/imagej/ (accessed on 30 June 2025)).

### 4.11. Public Databases Analysis

The prognostic significance of *RPS5*, ANT2 (encoded by *SLC25A5*), *ELOVL7*, *ELOVL6*, and *HADHB* expression in ER-positive, HER2-negative BC patients was evaluated using the Kaplan–Meier Plotter (https://kmplot.com/ (accessed on 30 June 2025)) [30]. The datasets utilized included GSE12093, GSE16391, GSE17705, GSE19615, GSE21653, GSE26971, GSE2990, GSE3494, GSE45255, GSE6532, and GSE9195. The “Auto select best cutoff” option was applied for stratification. Two patient cohorts were compared using Kaplan–Meier survival plots, with hazard ratios (HRs) and 95% confidence intervals (CIs) estimated, along with the log-rank test statistic and *p* value. To investigate the correlation between Luminal subtypes and the mRNA expression levels of *SLC25A5*, *ELOVL7*, and *ELOVL6*, data were obtained from the TCGA BC patient dataset on cBioPortal (https://www.cbioportal.org/ (accessed on 9 July 2025)) [28,29]. To compare the expression between normal and BC tissues, UALCAN (http://ualcan.path.uab.edu (accessed on 14 April 2025)) [25] was used to obtain *SLC25A5* mRNA expression data from 1211 cases in the TCGA database and ANT2 protein expression data from 143 cases in the CPTAC database. Using the TCGA BC dataset via the GEPIA online database (http://gepia.cancer-pku.cn/ (accessed on 25 June 2025)) [26], the correlations of *SLC25A5*, *ELOVL7*, and *ELOVL6* with the cell proliferation marker Ki-67 (coded by the *MKI67* gene) were analyzed. To compare *ELOVL7* and *ELOVL6* expression between responders and non-responders to endocrine therapy in ER-positive and HER2-negative BC, ROC Plotter (https://rocplot.com/ (accessed on 28 May 2025)) was used to analyze publicly available transcriptomic and treatment response data.

### 4.12. RNA Extraction and RNA-Sequencing Preprocessing

Total RNA was extracted using Sepasol^®^-RNA Super G (Nacalai Tesque). RNA quality was confirmed, and libraries were prepared using the NEBNext Poly(A) mRNA Magnetic Isolation Module and the NEBNext Ultra II Directional RNA Library Prep Kit (New England Biolabs, Ipswich, MA, USA). RNA sequencing (RNA-seq) was outsourced to Rhelixa Inc. (Tokyo, Japan). Sequencing was performed on an Illumina NovaSeq 6000 platform to generate paired-end 150 bp reads (PE150), yielding on average 26.7 million read pairs per sample (approximately 8.0 Gb bases, 2 × 150 bp × 26.7 M pairs). Raw read quality was assessed using FastQC v0.12.1, and adapter and low-quality sequences were trimmed using Trimmomatic v0.38. Trimmed reads were aligned to the human reference genome hg38 (GRCh38) using HISAT2 v2.1.0, and SAM files were converted into BAM format with Samtools v1.9. Gene-level counts were obtained using featureCounts v1.6.3 with the corresponding GTF annotation. Sequencing data were deposited into the DNA Data Bank of Japan Sequence Read Archive (accession nos. DRR793678 to DRR793681, DRR793798 to DRR793803).

### 4.13. RNA-Seq Data Analysis

The TPM expression matrix was imported into iDEP v2.01 (http://bioinformatics.sdstate.edu/idep/ (accessed on 14 April 2025)) [53] for differential gene expression analysis between groups. Differentially expressed genes (DEGs) were defined as those with |log_2_ fold change| > 1 and FDR < 0.1. Functional enrichment analysis of DEGs was performed using Metascape (https://metascape.org (accessed on 14 April 2025)) [54], with particular focus on KEGG pathways.

### 4.14. Staining and Quantification of Lipid Droplets

Lipid droplets were stained with ShoyakuGreen (Tokyo Chemical Industry Co., Ltd., Tokyo, Japan) according to the manufacturer’s instructions. Briefly, cells were seeded on coverslips placed in 6-well plates and cultured under each experimental condition. For staining, the culture medium was replaced with fresh medium containing 1 µM ShoyakuGreen, and the cells were incubated at 37 °C for 30 min. Cells were then fixed with 4% paraformaldehyde for 10 min. Nuclei were stained with 2 µg/mL Hoechst 33342 (Dojindo), and coverslips were finally mounted on glass slides. Lipid droplet quantification was performed after imaging using a BZ-X800 analyzer (Keyence Corporation, Osaka, Japan).

### 4.15. In Silico Drug Screening

Predicted ANT2 structure was generated by SWISS-MODEL [55] using the crystal structure of mitochondrial ADP/ATP carrier from Bos taurus (PDB ID: 1okc) as a template. The predicted structure of ANT2 was protonated and placed in a palmitoyl-oleoyl-phosphatidylcholine (POPC) lipid bilayer. The molecules were placed in a simulation box with dimensions of 8.21 nm × 8.21 nm × 10.84 nm, which was filled with water molecules. The simulation box was created using CHARMM-GUI [56]. The lipid molecules were done using the lipid21 force field [57]. The 100 ns simulation at 303.15 K was performed to generate a relaxed structure of ANT2. The relaxed ANT2 structure was used for molecular docking calculations. Existing drugs described in isomeric SMILES were derived from the PubChem, which satisfy the following criteria: (1) approved by the FDA, (2) molecular weight is less than 1000. The protonation state of drugs was predicted by dimorphite-DL [58]. The three-dimensional structures of drugs were generated by RDKit (https://www.rdkit.org (2023.9.5 version)). To create complex structures, molecular docking simulations were performed using Smina [31], and 10 docking poses were generated for each drug. The docking grid box was defined around the ATP/ADP-binding pocket [24]. MD simulation was performed to relax the docking pose. The docking structure of ANT2 and each drug was protonated and placed in a dodecahedral box. The box size was determined to ensure that all molecules were placed at least 1.5 nm from the box edges. The periodic boundary conditions were applied in all directions. The box was filled with water molecules. Sodium or chloride ions were added to the box to neutralize the total charge. The ANT2 molecule was described using the AMBERff14SB force field [59]. The drug molecules were described using the GAFF force field [60] with restrained electrostatic potential (RESP) charges [61]. Water molecules were described using the TIP3P model [62], and ion parameters were taken from the model developed by Joung and Cheatham [63]. The temperature was maintained at 303.15 K using Langevin dynamics, and a pressure of 1 atm was maintained using Parinello–Rahman barostat [64]. The MD simulations were performed using GROMACS 2022.4 [65]. These structures were then evaluated based on the binding affinity predicted by Smina’s scoring function [31], and the most stable docking pose was selected. Each drug’s score determined its ranking among the candidate drugs. Finally, the chosen candidate drugs for the subsequent validation study were selected from the high-ranking medicines, based on their availability and therapeutic utility. The structure images were generated using PyMOL (v3.1.6.1, Schrödinger, LLC, New York, NY, USA).

### 4.16. Statistical Analysis

All data are presented as the mean ± standard deviation (SD). The significance of differences in the means among three or more groups was analyzed using a one-way analysis of variance (ANOVA). In comparison, comparisons between two groups were tested using a two-tailed unpaired Student’s *t*-test. *p* values < 0.05 were considered significantly different from the controls. Survival curves were generated using the Kaplan–Meier method, and the statistical significance of differences between groups was assessed with the log-rank test; HRs and 95% CIs were calculated.

## Data Availability

All the sequencing raw data were deposited in the DNA Data Bank of Japan Sequence Read Archive (accession nos. DRR793678 to DRR793681, DRR793798 to DRR793803).

## Supplementary Materials

The following supporting information can be downloaded at: https://www.mdpi.com/article/10.3390/ijms27083704/s1.
