# Supplementary material for: Identification of ANT2 as a Druggable Target for Endocrine-Resistant ERα-Positive Breast Cancer
Source: Int J Mol Sci. 2026 Apr 21;27(8):3704. doi: 10.3390/ijms27083704 (PMC13116172; doi:10.3390/ijms27083704)
Supplement: Supplementary file 1 [file ijms-27-03704-s001.zip › Supplymental Information.pdf]

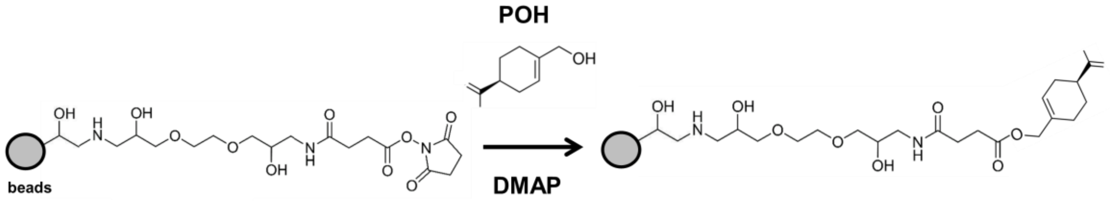


**Figure S1. Scheme for fixation of POH onto magnetic FG beads.** Immobilized POH on magnetic FG beads and the estimated structure of beads harboring the compound.

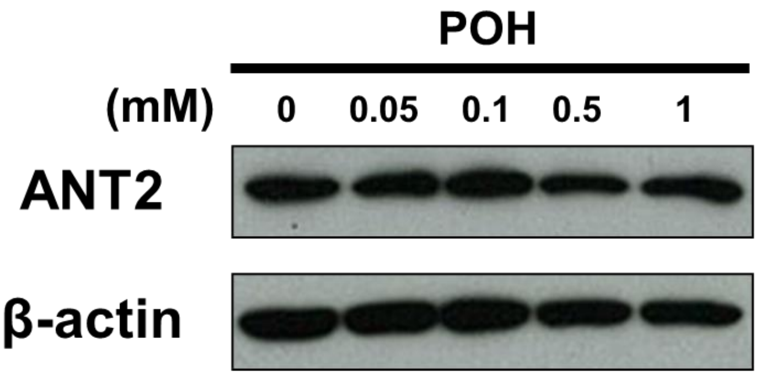


**Figure S2. POH had no effect on ANT2 expression levels in ER-positive BC cells.** Analysis of the expression of ERα protein after the dose-dependent treatment of POH. MCF7 cells treated with POH at the indicated concentrations for 24 hr. β-Actin was used as a loading control.

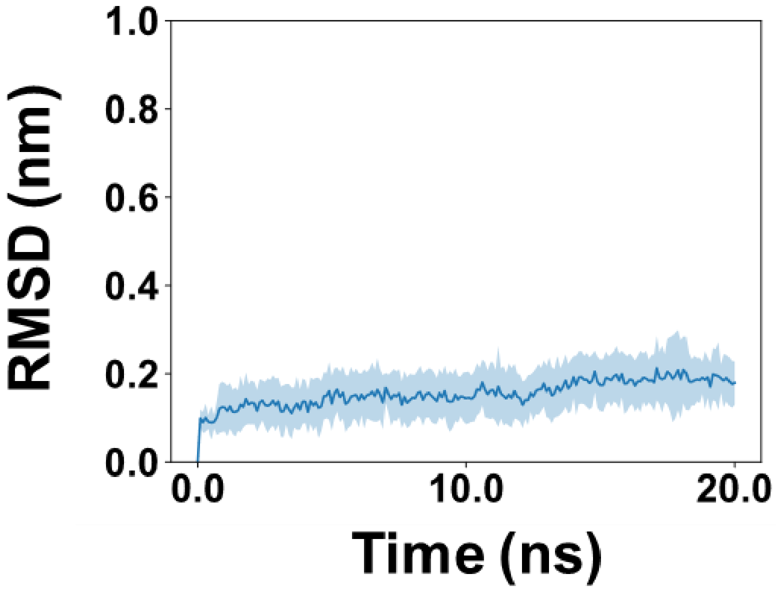


**Figure S3. RMSD values of POH from the initial conformation.** RMSD values indicate the deviation from the initial positions in 20 ns × 10 simulations. Solid line: mean; shaded area: standard deviation.

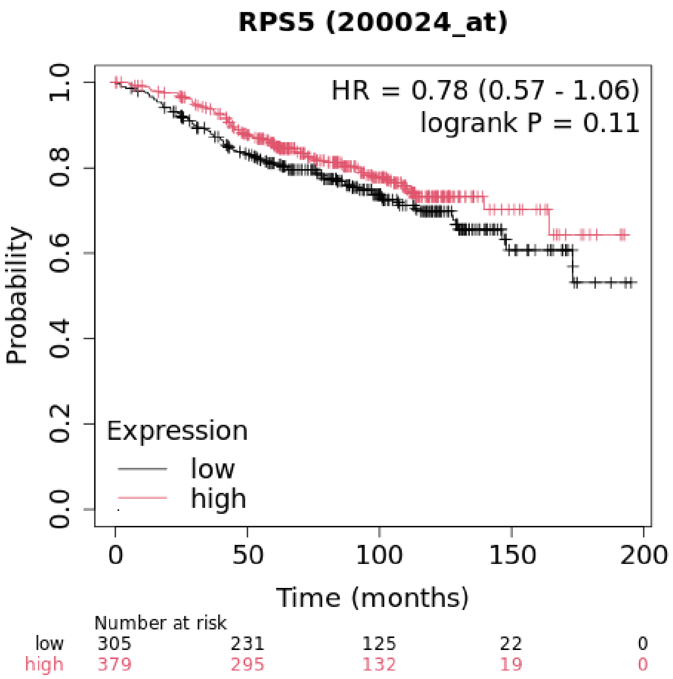


**Figure S4. High expression of *RPS5* was not associated with prognosis.** Prognosis analysis of ER-positive BC patients treated with endocrine therapy. Patients were stratified by *RPS5* expression, and recurrence-free survival (RFS) was analyzed using Kaplan-Meier Plotter (https://kmplot.com/).

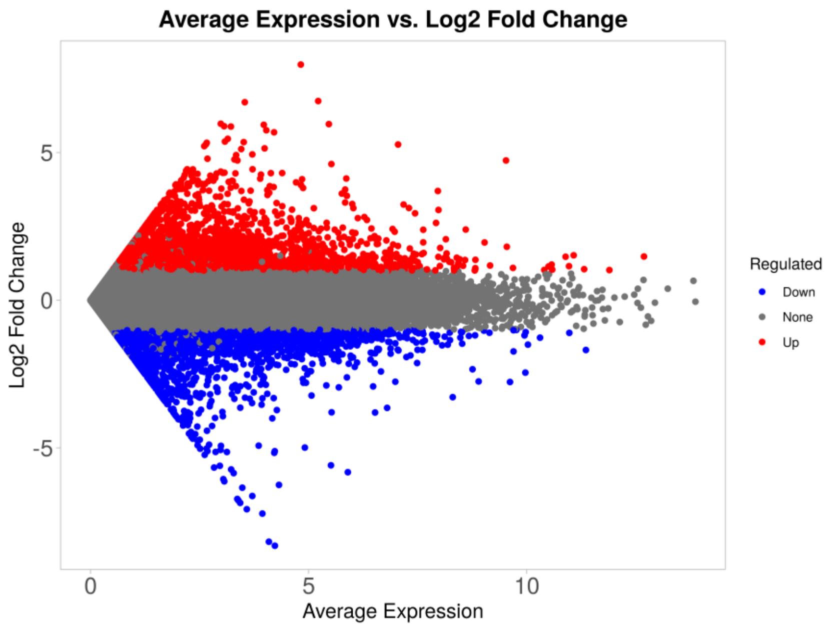


**Figure S5. Differential gene expression analysis between MCF7 cells and 182R-1 cells.** In the MA plot, genes upregulated and downregulated in 182R-1 cells compared with MCF7 cells are indicated in blue and red, respectively.

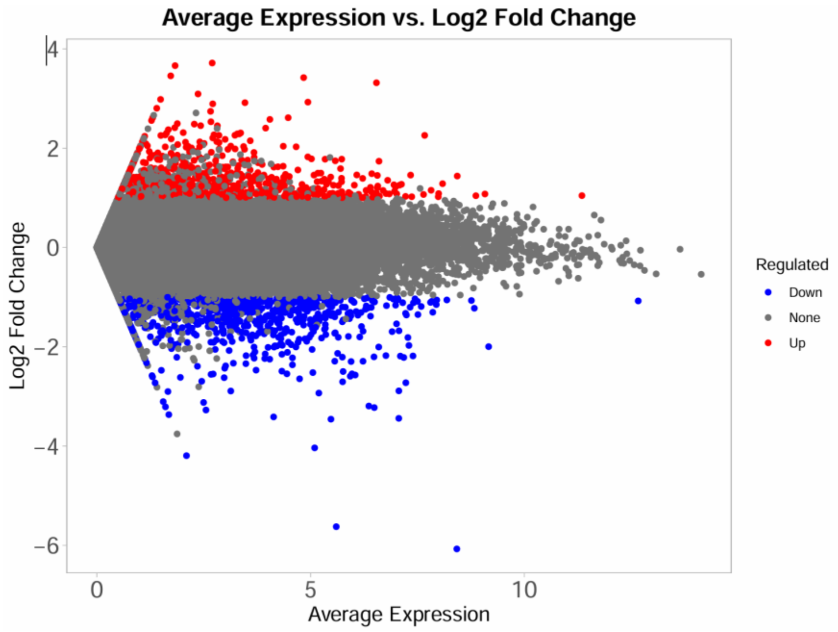


**Figure S6. Differential gene expression analysis between 182R-1 cells after siANT2 transfection and those of the negative control siRNA.** In the MA plot, upregulated and downregulated genes in ANT2-depleted 182R-1 cells are indicated in blue and red, respectively.

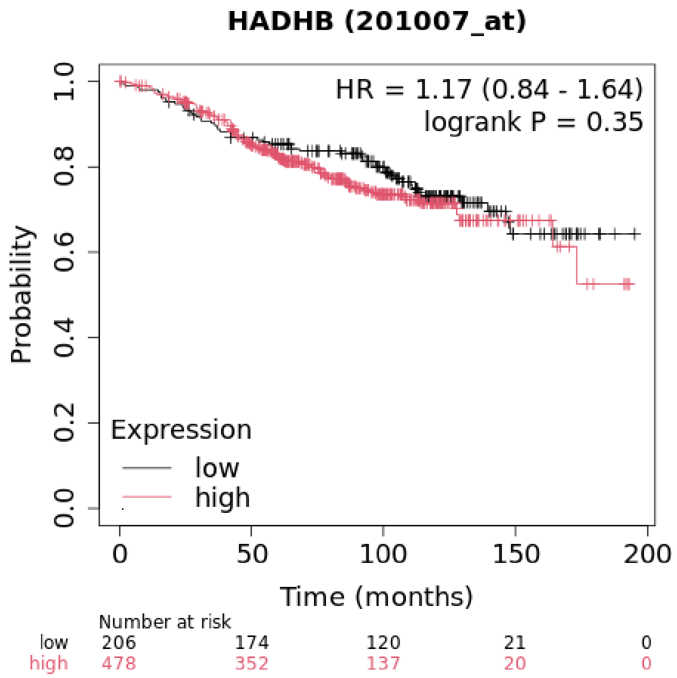


**Figure S7. High expression of *HADHB* was not associated with prognosis.** Prognosis analysis of ER-positive BC patients treated with endocrine therapy. Patients were stratified by *HADHB* expression, and recurrence-free survival (RFS) was analyzed using Kaplan-Meier Plotter (https://kmplot.com/).

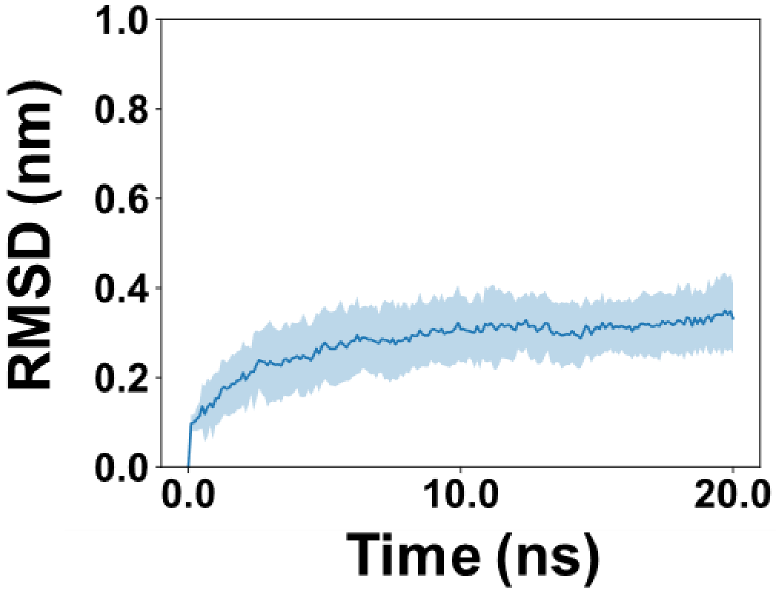


**Figure S8. RMSD values of Venetoclax from the initial conformation.** RMSD values indicate the deviation from the initial positions in 20 ns × 10 simulations. Solid lines: mean; shaded areas: standard deviation.

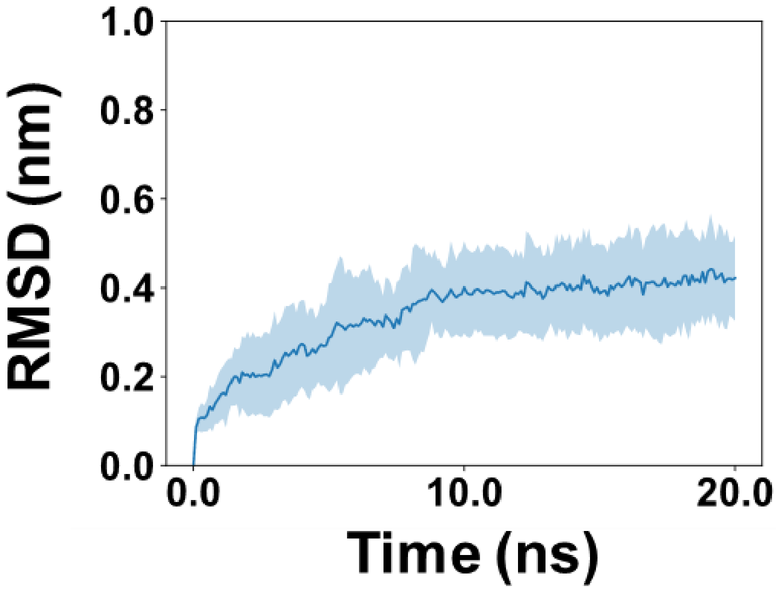


**Figure S9. RMSD values of Nystatin A1 from the initial conformation.** RMSD values indicate the deviation from the initial positions in 20 ns × 10 simulations. Solid lines: mean; shaded areas: standard deviation.

| siRNA Name | Target Nucleotide Sequence (5'-3') | Target Gene |
|---|---|---|
| siANT2 #1 | AGAACAUUGGACCAUGCACCCUUGA | ANT2 |
| siANT2 #2 | UGUAUUGCUUAUCUGCAGUGAUCUG | ANT2 |
| siRPS5 #1 | GGAGCACCGAUGAUGUGCAGAUCAA | RPS5 |
| siRPS5 #2 | CCUGCAGGAUUACAUUGCAGUGAAG | RPS5 |

**Table S1. siRNA sequences targeting ANT2 and RPS5 used in this study.**

**Video S1. Molecular dynamics (MD) trajectory of the ANT2-POH complex.**

**Video S2. Molecular dynamics (MD) trajectory of the ANT2-Venetoclax complex.**

**Video S3. Molecular dynamics (MD) trajectory of the ANT2-Nystatin A1 complex.**
